# Tetrandrine Inhibits Skeletal Muscle Differentiation by Blocking Autophagic Flux

**DOI:** 10.3390/ijms23158148

**Published:** 2022-07-24

**Authors:** Jing Li, Meiyun Shi, Lutao Liu, Jiahui Wang, Minsheng Zhu, Huaqun Chen

**Affiliations:** 1 Jiangsu Key Laboratory for Molecular and Medical Biotechnology, College of Life Sciences, Nanjing Normal University, Nanjing 210023, China; 15651729189@163.com (J.L.); 18625167990@163.com (M.S.); nectarinellt@gmail.com (L.L.); 18652932281@163.com (J.W.); 2 State Key Laboratory of Pharmaceutical Biotechnology, Medical School of Nanjing University, Nanjing 210008, China; zhums@nju.edu.cn

**Keywords:** tetrandrine, autophagy, myoblast, mitochondria, myogenic differentiation

## Abstract

Tetrandrine is well known to act as a calcium channel blocker. It is a potential candidate for a tumor chemotherapy drug without toxicity. Tetrandrine inhibits cancer cell proliferation and induces cell death through apoptosis and autophagy. As cancer patients usually experience complications with sarcopenia or muscle injury, we thus assessed the effects of tetrandrine on skeletal muscle cells. We report in this study that a low dose of tetrandrine (less than 5 μM) does not affect the proliferation of C2C12 myoblasts, but significantly inhibits myogenic differentiation. Consistently, tetrandrine inhibited muscle regeneration after BaCl_2_-induced injury. Mechanistic experiments showed that tetrandrine decreased the p-mTOR level and increased the levels of LC3 and SQSTM1/p62 during differentiation. Ad-mRFP-GFP-LC3B transfection experiments revealed that the lysosomal quenching of GFP signals was suppressed by tetrandrine. Furthermore, the levels of DNM1L/Drp1, PPARGA1 and cytochrome C (Cyto C), as well as caspase 3 activation and ROS production, were decreased following tetrandrine administration, indicating that the mitochondrial network signaling was inhibited. Our results indicate that tetrandrine has dual effects on autophagic flux in myoblasts during differentiation, activation in the early stage and blockade in the late stage. The ultimate blocking of autophagic flux by tetrandrine led to the disruption of mitochondria remodeling and inhibition of myogenic differentiation. The inhibitory effects of tetrandrine on skeletal muscle differentiation may limit its application in advanced cancer patients. Thus, great attention should be paid to the clinical use of tetrandrine for cancer therapy.

## 1. Introduction

In human adults, the loss of skeletal muscle accompanies general physiological activities. Progenitors in skeletal muscle can proliferate and differentiate into myotubes, and the myotubes can fuse with the existing myofibers and thereby replenish muscle mass [1,2]. C2C12 myoblasts are a well-known immortal skeletal muscle progenitor cell line, originally derived from mouse satellite cells, which can differentiate into myotubes in vitro [3].

Tetrandrine is a natural product isolated from the plant *S. tetrandra S. Moore* [4]. Tetrandrine belongs to the bisbenzylisoquinoline alkaloid family and has been reported to be a potent calcium channel blocker that inhibits voltage-gated Ca^2+^ channels [5,6]. Recently, it was documented that in multiple cancer cells, the acidity of lysosomes was reduced by tetrandrine, resulting in the blockade of autophagic flux [7]. This activity may not be related to the change in intracellular calcium. A number of studies have demonstrated that tetrandrine has pharmacological potential as an antitumor chemotherapeutic drug with a broad range of bioactivities, including inhibiting cellular proliferation, modulating cell metabolism and motility, trigging reactive oxygen species (ROS) accumulation and cell death by activating autophagy at low concentrations and inducing apoptosis at high concentrations [8,9]. Interestingly, tetrandrine facilitates megakaryocyte differentiation in leukemic cells by activating autophagy [10].

Autophagy selectively removes unnecessary, damaged or dysfunctional proteins and organelles. This is an essential biological process in the transition from myoblasts to myotubes, eliminating dysfunctional proteins and organelles through the lysosome degradation pathway [11]. During differentiation, mitochondria and other organelles are recruited by autophagy receptors, such as SQSTM1/p62, to form double-membrane vesicles known as autophagosomes, which are indicated by the lipid-conjugated form of LC3, LC3-II [12]. Then, mature autophagosomes fuse with lysosomes and form autolysosomes, the cargo is digested by lysosomal enzymes, and the resulting molecules are recycled back to the cytoplasm. The degradative activity of the enzymes requires the acidified environment of the lysosome. Weak bases, such as chloroquine (CQ), an antimalarial drug and a promising cancer treatment drug, can be protonated and accumulate in lysosomes, resulting in the deacidification of lysosomal lumens and blocking autophagic flux [13,14].

Tetrandrine is well known to act as a calcium channel blocker. It is a potential candidate for anticancer chemotherapy [9]. It has been tested in clinical trials and found effective against silicosis, hypertension, inflammation and lung cancer without any toxicity [15]. There is a report showing a beneficial effect of tetrandrine on reversing human cardiac myofibroblast activation and myocardial fibrosis through a mechanism-independent calcium channel blockade [16]. Cancer patients usually experience complications with sarcopenia and muscle injury [17,18,19]. Whether tetrandrine also effects skeletal muscle is an interesting issue and knowledge about this would be very useful in the use of this drug in clinical trials. In the present study, we examined the effects of tetrandrine on the proliferation and myogenic differentiation of myoblasts and the underlying mechanisms. Our findings indicate that a low concentration of tetrandrine (≤5 μM) is nontoxic to the growth of myoblasts but can inhibit myogenesis progression by blocking autophagic flux and disturbing mitochondrial network signaling during differentiation. Our results suggest that great attention should be paid to the clinical use of tetrandrine for cancer therapy, particularly for patients with sarcopenia.

## 2. Results

### 2.1. Low-Dose Tetrandrine Is Sufficient to Inhibit Myoblast Differentiation

**Figure 1:**
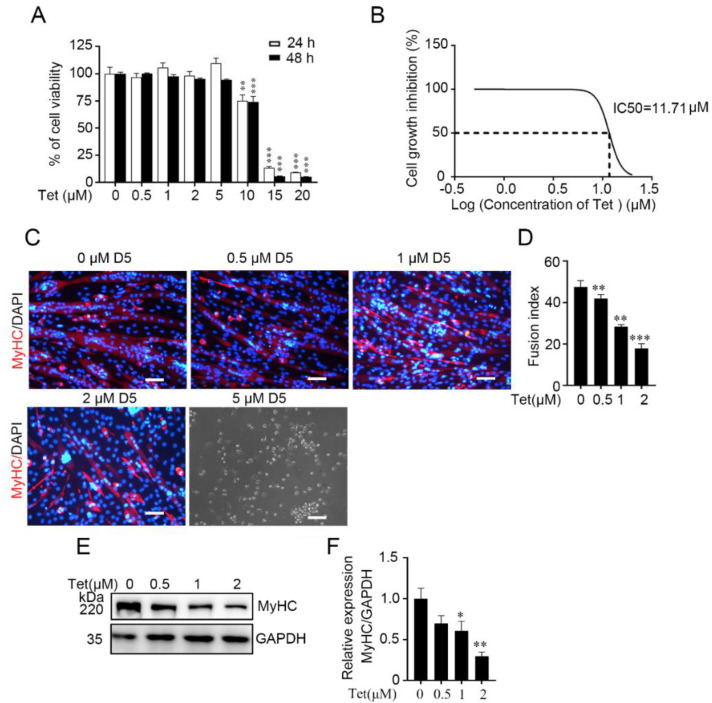
Tetrandrine inhibits myogenic differentiation in C2C12 myoblasts. (**A**): An MTT assay was performed to measure the cytotoxic effect of tetrandrine on C2C12 myoblasts. *n* = 3, ** *p* < 0.01 and *** *p* < 0.001. (**B**): The IC50 of tetrandrine on C2C12 cells was calculated. (**C**): C2C12 myoblasts were differentiated for 5 d in DM with or without tetrandrine and immunostained with anti-MyHC (MF20) (red). The nuclei were counterstained with DAPI (blue). (**D**): The fusion index in C was determined. *n* = 3, ** *p* < 0.01 and *** *p* < 0.001. Scale bars: 50 μm. (**E**): Representative Western blots showing the expression of MyHC in C2C12 myoblasts after 5 days of differentiation (D5). (**F**): The relative expression of MyHC in E was quantified. *n* = 3, * *p* < 0.05 and ** *p* < 0.01. Tet, tetrandrine; MyHC, myosin heavy chain; GAPDH, Glyceraldehyde-3-phosphate dehydrogenase; IC50, half maximal inhibitory concentration.

To determine the effect of tetrandrine on skeletal muscle differentiation, we used the C2C12 myoblast cell line as an in vitro model of myogenesis. Because tetrandrine is toxic to mammalian cells, particularly cancer cells [9,20], we first measured its dose-responsive effect on C2C12 proliferation. When the concentration of tetrandrine was lower (up to 5 μM), no alteration in C2C12 proliferation/viability was detected by MTT assays (Figure 1A). The IC50 value was 11.71 μM (Figure 1B). Therefore, we used tetrandrine at concentrations less than 5 μM in our subsequent experiments.

After differentiation medium was added, C2C12 cells began to differentiate and fused to form typical myotubes. The immunofluorescence staining results showed that the newly formed myotubes were smaller in the presence of tetrandrine (0.5 to 2 μM) on day 5 compared with that in the DMSO control group. Consistently, the fusion index was decreased (*p* < 0.01) (Figure 1C,D). These data indicated that a low dose of tetrandrine (2 μM) was sufficient to inhibit myogenic differentiation of C2C12 myoblasts. In addition, no apparent cell death was observed during differentiation in the presence of a low concentration of tetrandrine (≤2 μM). However, most of the cells were dead and floating after being exposed to the higher concentration of tetrandrine (5 μM). These results indicate that a low concentration of tetrandrine (≤2 μM) can inhibit C2C12 differentiation, and this effect is unlikely to be due to general cytotoxicity. Western blotting consistently showed that the expression of MyHC was markedly decreased (Figure 1E,F).

To further investigate this effect, we cultured primary myoblast cells from C57BL/6 mice and repeated this experiment. As expected, the differentiation of primary myoblasts was also inhibited by tetrandrine (Figure S1), but the inhibition was less potent in contrast to that of C2C12 cells. In the presence of 2 µM tetrandrine, C2C12 cells (D5) showed a 62.4% decrease in differentiation index (from 47.51 ± 3.04% to 17.89 ± 2.26%), while the primary myoblasts (D5) showed a 79.3% decrease (from 29.66 ± 2.32% to 6.13 ± 0.82%). This difference might be due to the intrinsic differences between the immortal cell line and primary culture cells. This difference was observed also by other investigators [21,22].

### 2.2. Tetrandrine Inhibits Skeletal Muscle Regeneration In Vivo

**Figure 2:**
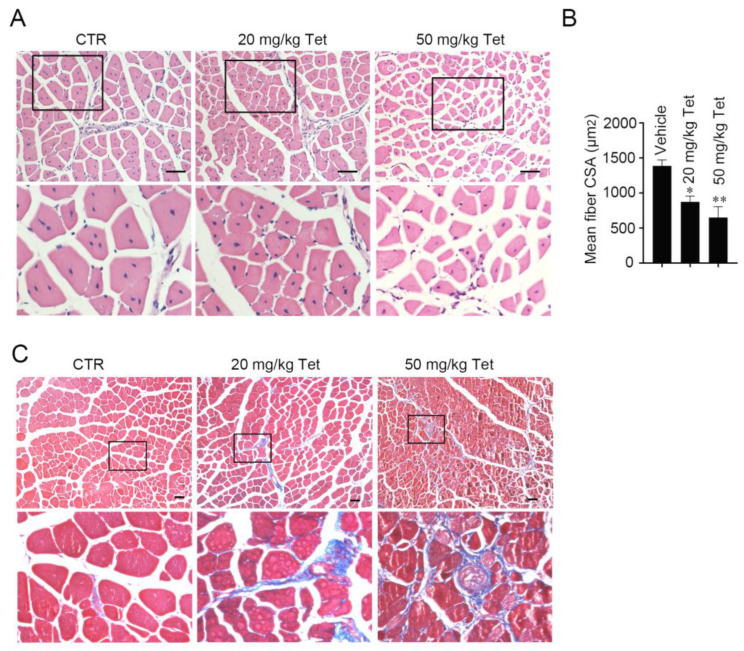
Tetrandrine inhibits skeletal muscle regeneration. Injury was induced in the left TA muscles of mice by intramuscular injection of BaCl2, and the mice were randomly distributed into three groups (five mice per group): vehicle and tetrandrine (20 and 50 mg·kg^−1^ body weight) P.O. every other day for 28 days. (**A**): H/E-stained cross-sections of the regenerated TA muscles. (**B**): The average cross-sectional area (CSA) (mean ± S.D.) of the muscle fibers in A was quantified. *n* = 5, * *p* < 0.05 and ** *p* < 0.01. (**C**): Masson’s trichrome-stained cross-sections of regenerated TA muscles and the fibrotic regions are shown (blue color). Scale bars: 50 μm. CTR, control mice; Tet, tetrandrine; CSA, cross-sectional area.

Given that tetrandrine has potent inhibitory activity toward skeletal muscle differentiation, tetrandrine could also attenuate regeneration after injury. To test this hypothesis, we examined the effect of tetrandrine on the repair of injured TA muscles. As expected, histological analysis showed that the mean CSA of the regenerated myofibers was significantly decreased in tetrandrine-treated groups compared with the vehicle control group (*p* < 0.05 for 20 mg·kg^−1^ tetrandrine vs. vehicle control; *p* < 0.01 for 50 mg·kg^−1^ tetrandrine vs. vehicle control) (Figure 2A,B) in a dose-dependent manner. Masson’s trichrome staining showed apparent fibrosis in the regenerated muscles of tetrandrine-treated mice (Figure 2C). These results indicate that tetrandrine can inhibit muscular regeneration in vivo.

### 2.3. Tetrandrine Increases Intracellular Autophagosomes during Myoblast Differentiation

**Figure 3:**
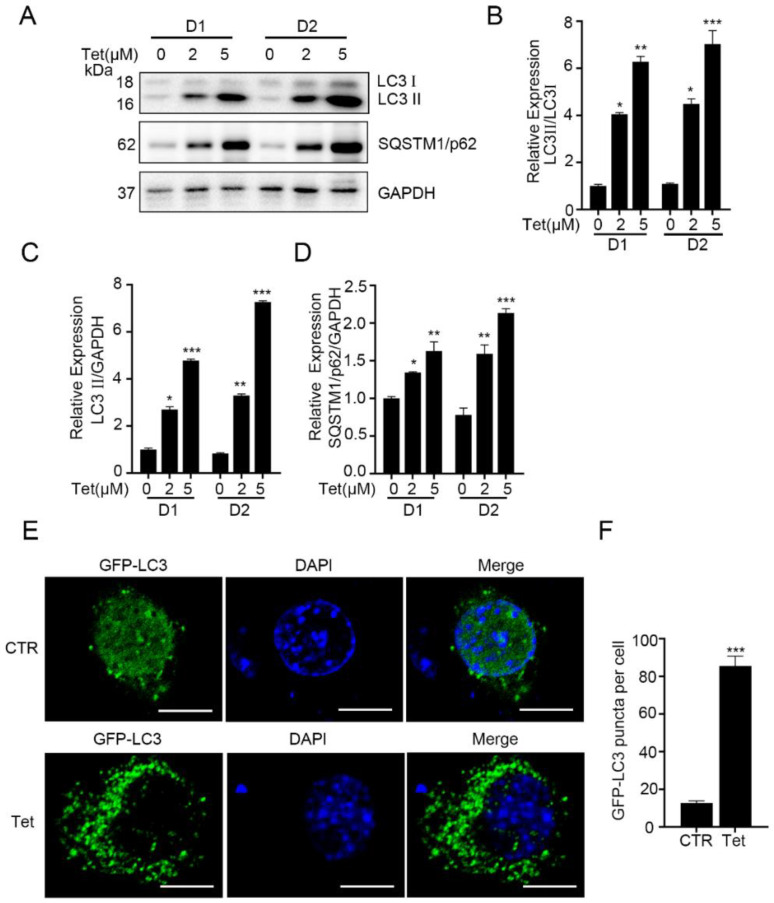
Tetrandrine induces autophagosome accumulation in differentiating C2C12 myoblasts. (**A**): Representative Western blots showing LC3 and SQSTM1/p62 levels in C2C12 myoblasts after 1 or 2 d of differentiation in the presence or absence of tetrandrine. (**B**–**D**): The expression ratios of LC3II/LC3I, LC3II/GAPDH and SQSTM1/p62/GAPDH in A were quantified using ImageJ 1.46r software (Bethesda, MD, USA) (https://imagej.nih.gov/ij, accessed on 29 June 2022). *n* = 3, * *p* < 0.05, ** *p* < 0.01 and *** *p* < 0.001. (**E**): GFP-LC3 puncta in C2C12 cells transfected with ad-GFP-LC3 and differentiated for 1 d in the presence or absence of tetrandrine. The nuclei were counterstained with DAPI (blue). Scale bars: 10 μm. (**F**): The numbers of GFP-LC3 puncta in E were quantified. *n* = 3, *** *p* < 0.001. LC3, microtubule-associated protein 3; SQSTM 1, sequestosome 1; CTR, vehicle-treated control cells; Tet, tetrandrine; GFP, green fluorescent protein.

To investigate the mechanism of the inhibitory effects of tetrandrine on myogenesis, we reviewed previous reports about tetrandrine and its association with myogenesis [10,23,24]. Because regulated autophagy levels are essential for myogenesis [25] and tetrandrine can efficiently activate autophagy in cancer cells [26], we hypothesized that tetrandrine could affect myogenesis by regulating autophagy. To address this hypothesis, we measured the expression levels of autophagy-related proteins in differentiating C2C12 myoblasts. In the absence of tetrandrine, differentiating C2C12 cells expressed relatively low protein levels of LC3 and SQSTM1/p62 and had a low LC3II/LC3I ratio (Figure 3A–D). However, in the presence of tetrandrine (2 μM and 5 μM), the LC3II/LC3I ratio and the expression levels of these proteins increased significantly (*p* < 0.05 and *p* < 0.01, 0.001, respectively). Furthermore, we showed that tetrandrine could cause the accumulation of autophagosomes in C2C12 myoblasts during differentiation (Figure 3E,F). C2C12 cells were transfected with ad-GFP-LC3 and induced to differentiate in the presence of tetrandrine. Significantly more GFP-LC3 puncta were observed in 2 μM tetrandrine-treated cells on D1 than in the control cells (*p* < 0.001).

### 2.4. Tetrandrine Blocks the Degradation of Autophagosomes in Differentiating C2C12 Myoblasts

**Figure 4:**
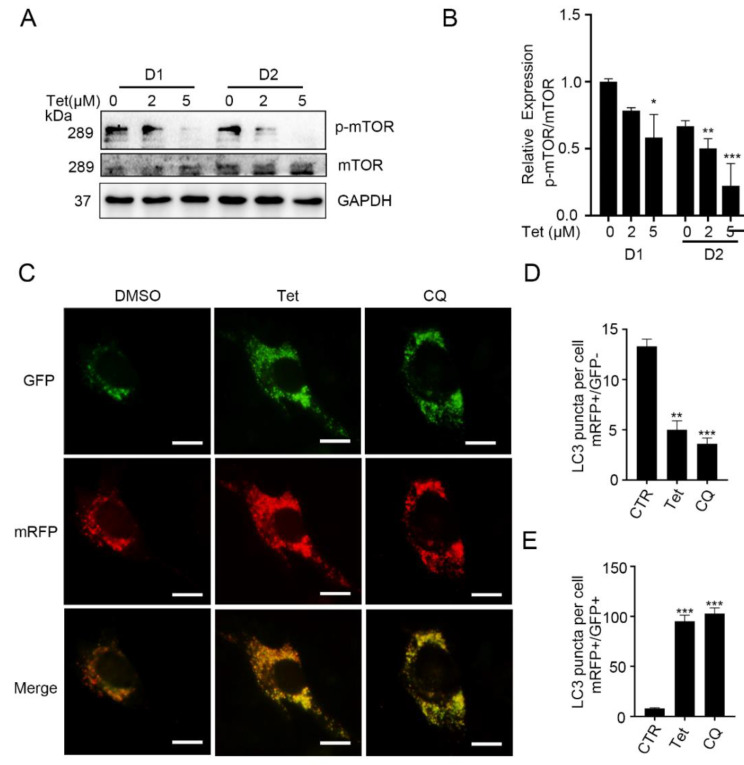
Tetrandrine activates the early stage and blocks the late stage of differentiation-induced autophagy. (**A**): Representative Western blots showing p-mTOR and mTOR levels in C2C12 myoblasts after 1 or 2 d of differentiation in the presence or absence of tetrandrine. (**B**): The expression ratios of p-mTOR/mTOR in A were quantified using Image J. *n* = 3, * *p* < 0.05, ** *p* < 0.01 and *** *p* < 0.001. (**C**): Fluorescent images of ad-mRFP-GFP-LC3-transfected C2C12 myoblasts after 1 d of differentiation in the presence of tetrandrine (2 μM) or CQ (10 μM). (**D**,**E**): The numbers of degraded autophagosomes (RFP+/GFP− puncta per cell) and undegraded autophagosomes (mRFP+/GFP+ puncta per cell) in (**C**) were quantified. *n* = 3, ** *p* < 0.01 and *** *p* < 0.001. Scale bars: 10 μm. Tet, tetrandrine; DMSO, dimethyl sulfoxide; GFP, green fluorescent protein; mRFP, membrane-localized red fluorescent protein; CQ, chloroquine.

The increases in LC3 and LC3II/LC3I could be attributed to the activation of the early stage of autophagic flux and the blockade of the late stage of autophagic flux [27]. Tetrandrine has been reported to induce autophagy in an mTOR-dependent manner, as well as to inhibit the lysosomal degradation of autophagosomes in cancer cells [7,26]. We thereby evaluated the effects of tetrandrine on differentiating C2C12 cells. As shown in Figure 4A,B, the p-mTOR levels were significantly inhibited in the presence of tetrandrine on D1 and D2, indicating that differentiation-induced autophagy was activated at the early stage.

To determine whether tetrandrine modulates autophagosome degradation, we introduced mRFP-GFP-LC3 into C2C12 cells and induced differentiation. We observed that on D1, the number of red puncta but not green puncta (mRFP+/GFP−, representing degraded autolysosomes) was significantly lower in the presence of tetrandrine than in the DMSO control group (*p* < 0.01). In contrast, the number of yellow puncta (mRFP+/GFP+, representing undegraded autophagosomes) was significantly higher in tetrandrine-treated cells than in DMSO control cells (*p* < 0.001). Similar results were observed in CQ-treated cells (Figure 4C–E). According to the principles of the mRFP-GFP assay, GFP signals can be quenched under acidic conditions (pH < 5) inside the lysosome lumen, and mRFP signals show no significant changes [28,29]. Therefore, our results also suggest that tetrandrine may cause a rise in pH in the lysosomes in differentiating C2C12 myoblasts and block the autophagic flux.

### 2.5. Tetrandrine Suppresses Caspase 3 Activation, ROS Generation and Mitochondrial Remodeling during Myogenic Differentiation

**Figure 5:**
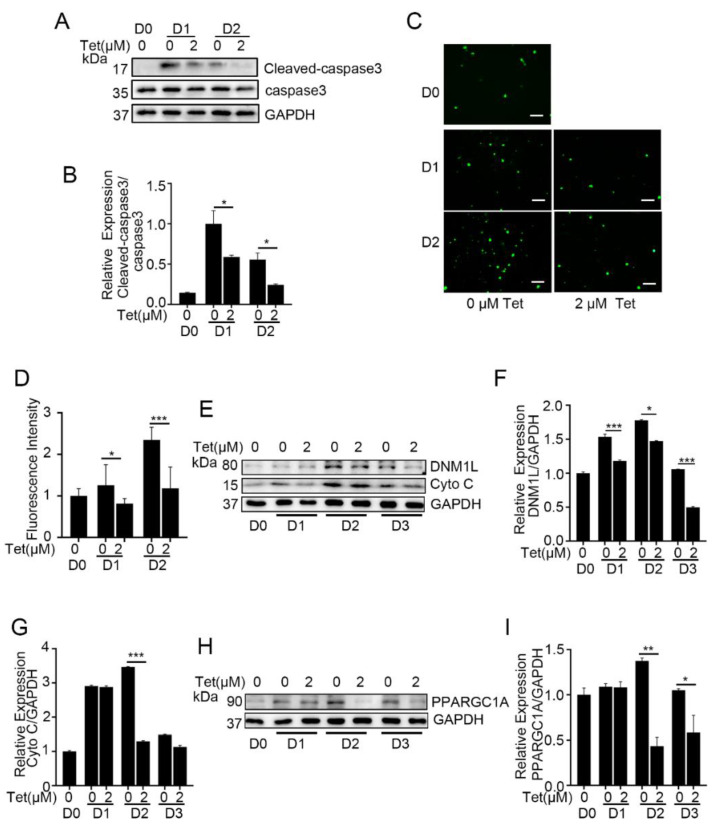
Tetrandrine inhibits mitochondrial remodeling in C2C12 cells during differentiation. (**A**): Representative Western blots showing cleaved caspase 3 and caspase 3 in C2C12 cells after 1 d and 2 d of differentiation. (**B**): Relative expression of cleaved caspase 3 and caspase 3 in A was quantified. *n* = 3, * *p* < 0.05. (**C**): H2DCF-DA detected ROS signals in C2C12 myoblasts during differentiation (0, 1 and 2 d) in the presence or absence of tetrandrine (2 μM). Scale bars: 50 μm. (**D**): The relative fluorescence intensity was quantified in (**C**). *n* = 3, * *p* < 0.05 and *** *p* < 0.001. (**E**): Representative Western blots showing DNM1L/Drp1 and Cyto C in C2C12 cells after 1 and 2 d of differentiation. (**F**,**G**): The relative expression of DNM1L/Drp1 and Cyto C in E was quantified. *n* = 3, * *p* < 0.05 and *** *p* < 0.001. (**H**): Representative Western blots showing PPARGC1A in C2C12 cells after 1 and 2 d of differentiation. (**I**): The relative expression of PPARGC1A in H was quantified. *n* = 3, * *p* < 0.05 and ** *p* < 0.01. Tet, tetrandrine; ROS, reactive oxygen species; Cyto C, cytochrome C.

Tetrandrine-induced blockade of autophagic flux was reported to mediate caspase 3 activation and apoptosis in tumor cells [7]. Moreover, autophagy inhibition enhanced caspase 9/caspase 3 activity in differentiating C2C12 myoblasts and impaired myogenesis [11]. Thus, we measured caspase 3 activation in tetrandrine-treated C2C12 cells. The data showed that differentiation-induced caspase 3 activation was significantly suppressed by tetrandrine administration in C2C12 myoblasts on D1 and D2 (*p* < 0.05) (Figure 5A,B). These results indicated the inhibition of mitochondria-related apoptosis, and we then measured the ROS levels of these cells because mitochondria are considered the main source of ROS production [30] As expected, ROS production declined in C2C12 cells on D1 and D2 in the presence of tetrandrine (*p* < 0.05) (Figure 5C,D).

Mitochondrial remodeling and autophagy are critical for skeletal muscle differentiation and myotube formation [31,32], and we next examined mitochondrial network-related factors in differentiating C2C12 myoblasts. The data showed that the levels of the markers of mitochondrial fission (DMN1L/Drp1), biogenesis (PPARGC1A) and content (cytochrome C, Cyto C) [33,34] were decreased following tetrandrine administration (Figure 5D–I), indicating that mitochondrial remodeling, including fragmentation and reconstitution, was inhibited by tetrandrine.

## 3. Discussion

Tetrandrine has been widely used to treat several diseases, including rheumatoid arthritis, sepsis, silicosis, hypertension and tumors [9,35,36,37,38]. Recent findings reveal that tetrandrine regulates autophagy [7], suggesting that pathophysiological processes are affected by tetrandrine. In this study, we found a novel role of tetrandrine in skeletal muscle. Low concentrations of tetrandrine, which are not toxic to cells, significantly inhibited myogenic differentiation in vitro and in vivo (Figure 1 and Figure 2).

It has been shown that tetrandrine can upregulate the expression of autophagy proteins, such as LC3 I/II and SQSTM1/p62, in cancer cells, suggesting that it can activate autophagy [7,23,26,39]. There have been reports show that tetrandrine can also inhibit autophagy by blocking the late stage of autophagic flux, which is driven by the deacidification of lysosomes [7]. Based on our observations, during the differentiation of myoblasts, tetrandrine may affect both the early stage and the late stage of autophagy, finally showing an inhibition effect on autophagy. Blocking the lysosomal degradation of autophagosomes leads to the inhibition of mitochondrial fragmentation and reconstitution, which are necessary for myogenic differentiation and myotube formation. The different effects of tetrandrine on autophagy appear to vary by cell type.

The acidification of the lysosomal lumen is critical for the degradation of autophagosomes, which is the final step of autophagic flux. The ad-mRFP-GFP-LC3 transfection results showed that GFP-LC3 signal quenching was blocked, similar to the effects of CQ (Figure 4C,D), suggesting that tetrandrine deacidifies the lysosomal lumen in differentiating C2C12 cells. Another study reported that CQ could potentiate the anti-leukemia properties of tetrandrine [10]. These observations and our findings suggest that tetrandrine may change the pH of lysosomes in both tumor cells and nontumor cells. We currently cannot properly explain how tetrandrine treatment caused the deacidification of lysosome. However, as the influx of protons mediated by proton-pumping ATPase in lysosomes contributes to the acidification of lysosomes, which is accompanied by the generation of transmembrane voltage, and the cation channels also permeable to calcium may be involved in the dissipate of this voltage, we speculate that other channels, such as TPC and TRPML1, might be involved in tetrandrine-induced lysosome acidification [40,41,42].

**Figure 6:**
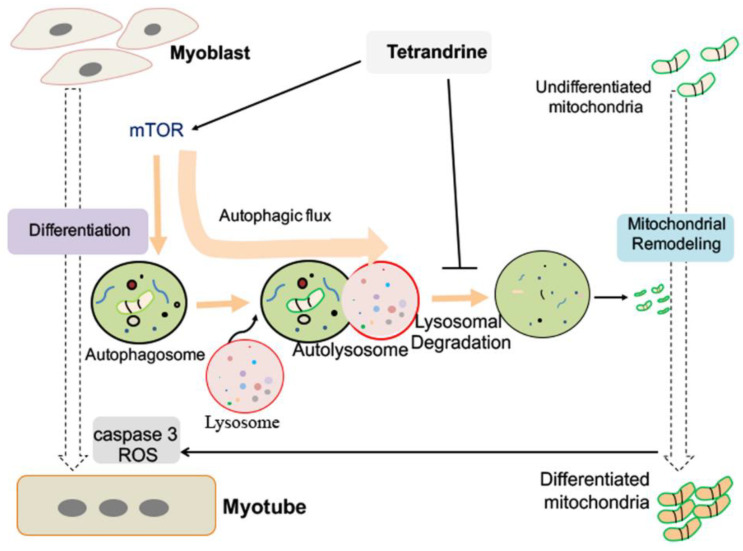
A working model for tetrandrine in myogenic differentiation of myoblasts. Tetrandrine activates the early stage and blocks the late stage of autophagic flux during the differentiation progression of myoblasts. The blockade of autophagic flux contributes to mitochondrial remodeling, which leads to a decrease in ROS production and caspase 3 activation, and thereby the inhibition of myogenic differentiation.

Autophagy is upregulated and required for myogenic differentiation. Previous studies have shown that 3-MA treatment and knockdown of ATG-7 inhibit autophagy and myogenesis, and increase caspase9/caspase 3 activity as well [11,31]. Here, we observed that tetrandrine-induced autophagy inhibition also inhibited myogenic differentiation. However, this effect was mediated by the suppression rather than enhancement of caspase 3 activation (Figure 5A), in contrast to the effect of 3-MA. The discrepant effects of these reagents on caspase 3 may be due to their distinct targets within the autophagy pathway. It has been well established that 3-MA inhibits the formation of autophagosomes, which is an early stage of autophagic flux [7], whereas tetrandrine both enhances the early stage and inhibits the late stage of autophagy, as we report here, ultimately blocking autophagic flux. This finding suggests that tetrandrine induces incomplete autophagic flux that prominently impairs mitochondrial network remodeling and myotube formation. This point is also supported by a previous study [32]. Similar roles and biological features of incomplete autophagic flux have been characterized in other biological processes, including cell death and cellular homeostasis [43]. In tumor cells, tetrandrine induced the blockade of autophagic flux, which resulted in incomplete autophagy and mitochondrial dysfunction, as revealed by the inhibition of ATP production and promotion of cell death [7]. However, in differentiating myoblasts, tetrandrine-induced blockade of autophagic flux disrupted the mitochondrial network, as shown by the decreases in caspase 3 activation and ROS generation (Figure 5A–D). The collective observations suggest multiple targets of tetrandrine that vary with the cell type and physiological process. A working model of tetrandrine is proposed in Figure 6.

Calcium activity is critical for proper muscle differentiation [44]. However, it is unlikely that the inhibitory effect of tetrandrine on myogenic differentiation is attributed to the change in calcium channel activity, since neither T-type nor L-type VDCCs are expressed in undifferentiated C2C12 cells and primary cultured satellite cells [45]. Our data suggest that tetrandrine may modulate myogenic differentiation in a calcium channel blocker-independent manner.

## 4. Materials and Methods

### 4.1. Chemicals and Reagents

Tetrandrine [(1b)-6,6′,7,12-tetramethoxy-2,2′-dimethylberbaman] was purchased from Chengdu MUST Biotechnology Company Limited (Chengdu, China) and dissolved in DMSO at a concentration of 5 mM stock solution. CQ was purchased from MCE (Shanghai, China) and dissolved in DMSO at a concentration of 200 mM.

Myosin heavy chain (MyHC) (MF20) antibody was purchased from DSHB (Iowa city, IA, USA). LC3-I/II, SQSTM1/p62 and GAPDH antibodies were obtained from Abcam (Shanghai, China). Caspase3/cleaved caspase 3 antibodies and HRP-conjugated secondary antibodies were purchased from CST (Beverly, MA, USA). DNM1L/Drp1, PPARGA1 and cytochrome C (CYC) antibodies were obtained from Santa Cruz (Dallas, TX, USA). p-mTOR/mTOR antibodies were purchased from Sciben (Nanjing, China).

### 4.2. Cell Culture

C2C12 myoblasts were purchased from ATCC (Rockville, MD, USA) (kindly provided by Prof. Yubo Zhang of the Chinese Academy of Agricultural Sciences, Shenzhen). The cells were cultured in DMEM (Thermofisher) (Waltham, MA, USA) supplemented with 10% (*v*/*v*) fetal bovine serum (FBS) (Thermofisher) (Waltham, MA, USA)and 1% penicillin/streptomycin (Thermofisher) (Waltham, MA, USA) in a humidified incubator containing 5% CO_2_ at 37 °C.

### 4.3. Isolation and Culture of Primary Myoblasts

Skeletal muscle tissues were removed from the limbs of neonatal mice. The tissues were cut into small pieces and digested with the enzyme mixture (1.5 U/mL collagenase D) (Roche) (Basel, Switzerland), 2.5 mM CaCl_2_ and 2 U/mL Dispase (Roche) (Basel, Switzerland) in serum-free DMEM at 37 °C for 0.5–1 h. Ten milliliters of DMEM was added to the digested mixture and triturated prior to centrifugation at 1500× *g* for 10 min at room temperature. The pellets were resuspended in Ham’s F-10 nutrient mixture (Thermofisher) (Waltham, MA, USA) containing 20% FBS, bFGF (R&D) (New York, USA) (5 ng/mL) and 1% penicillin/streptomycin and plated in a 10 cm culture dish for 1 h. The unattached cells were harvested and cultured in 24-well culture plates that were precoated with Matrigel (Sigma-Aldrich) (St. Louis, MO, USA).

### 4.4. Myogenesis Induction

When the cell confluence reached approximately 80%, the cells were placed in DMEM containing 2% horse serum (Sangon) (Shanghai, China) and 1% penicillin/streptomycin (differentiation medium, DM) to induce myogenesis. The fusion index was calculated by measuring the ratio of the nuclei number in myotubes with two or more nuclei versus the total number of nuclei (myoblasts + myotubes) observed in five randomly chosen areas [46].

### 4.5. In Vivo Studies

Male C57BL/6 mice (8–10 weeks) were purchased from GemPharmatech (Nanjing, China). The mice were maintained at the Nanjing Normal University specific-pathogen-free-grade animal facility (Nanjing, China). The animal experiments were approved by the Experimental Committee of Nanjing Normal University (No: IACYUC-1903023; Approval date: 1 March 2019). The muscle injury model was established as described in our previous study [47]. Briefly, 30 µL of 1.2% BaCl_2_ (Sigma-Aldrich) (St. Louis, MO, USA) dissolved in saline was injected into the tibialis anterior (TA) muscle. One day after the injury, the animals were randomly divided into three groups. Each group of animals (five mice per group) was administered (P.O.) vehicle (0.5% carboxymethyl cellulose) (Sigma-Aldrich) (St. Louis, MO, USA). or two doses (20 and 50 mg·kg^−1^ body weight) of tetrandrine every other day. The doses of tetrandrine were determined according to previous studies [10,48,49]. After 28 days of treatment, the mice were killed, and the TA muscles were collected. The tissues were fixed in 4% paraformaldehyde (PFA) (Biosharp) (Hefei, China) overnight. After being dehydrated through a series of ethanol baths, the samples were embedded in paraffin wax, and 4 µm cross-sections were stained with hematoxylin and eosin (H&E) (Beyotime) (Shanghai, China) for general histological analysis or Masson’s trichrome (Solarbio) (Beijing, China) for collagen staining. The cross-sectional area (CSA) of the myofibers was quantified with Photoshop 2018 software (Adobe Systems Incorporated, U.S.) licensed to Nanjing University (five fields per sample).

### 4.6. MTT Assay

C2C12 myoblasts were seeded at a density of 1 × 10^4^/mL in 96-well plates (100 µL per well) and cultured overnight. The cells were treated with different concentrations of tetrandrine for 24 or 48 h. Ten microliters of MTT (5 mg/mL) (Sigma-Aldrich) (St. Louis, MO, USA) was added to each well and incubated for another 4 h. The medium in each well was removed, and 100 μL of DMSO was added and incubated at 37 °C for 30 min. The absorbance at 570 nm was measured using a microplate reader (Biotek Synergy) (Winooski, VT, USA) and the half maximal inhibitory concentration (IC50) was calculated.

### 4.7. mRFP-GFP-LC3 Assay

Membrane-localized red fluorescent protein and green fluorescent protein-tagged LC3 adenoviral vectors (ad-mRFP-GFP-LC3) were purchased from HanBio Technology (Shanghai, China). Adenoviral infection was performed according to the manufacturer’s instructions. Briefly, C2C12 cells were incubated with DM containing the adenoviruses (MOI: 10) for 12 h at 37 °C, followed by myogenesis induction. At 24 h post-differentiation, LC3 puncta were examined under a fluorescence microscope (Olympus, BX41) (Tokyo, Japan). The images were captured and processed with Photoshop 2018 software.

### 4.8. Autophagosome Observation

The cells were infected with ad-GFP-LC3 (HanBio) (Shanghai, China) (MOI: 10) and induced to differentiate. GFP-LC3 puncta were examined under a fluorescence microscope (Olympus, BX41) (Tokyo, Japan). The images were captured and processed with Photoshop 2018 software.

### 4.9. ROS Measurement

Cellular ROS were measured using an ROS assay kit (Sciben) (Nanjing, China) according to the manufacturer’s procedures. Briefly, differentiating C2C12 myoblasts were incubated with the fluorescence probe 2′,7′-dichlorofluorescein (H2DCF-DA) (10 μM) for 20 min at 37 °C and washed with PBS. Images were captured under a fluorescence microscope (Leica) (Wetzlar, Germany) (five fields per sample). To quantify the ROS signals, green fluorescence-positive cells in the images were manually counted.

### 4.10. Western Blotting

Protein samples were prepared according to our previous study [50]. The resultant samples were subjected to electrophoresis on 8% or 12% (wt/vol) polyacrylamide gels and transferred onto PVDF membranes (Millipore) (St. Louis, MO, USA). The membranes were blocked with 5% (wt/vol) skim milk and sequentially probed with specific primary antibodies and appropriate HRP-conjugated secondary antibodies. The signals were then visualized by Western ECL Substrate (Sciben) (Nanjing, China) and scanned with a Tanon-4500 gel imaging system (Shanghai, China). The intensities of the protein bands were quantified using ImageJ 1.46r software (Bethesda, MD, USA) (https://imagej.nih.gov/ij, accessed on 29 June 2022).

### 4.11. Statistical Analysis

The data were analyzed by GraphPad Prism 6.0 software (San Diego, CA, USA) (https://www.graphpad.com, accessed on 29 June 2022) and shown as mean ± SD. One-way ANOVA was used to calculate *p* values, followed by Dunnett’s post-hoc test. *p* < 0.05 was considered statistically significant.

## 5. Conclusions

Our study demonstrated that a low dose of tetrandrine has dual effects on differentiation-induced autophagy and the blockade of autophagic flux contributes to the disruption of mitochondrial network signaling, thereby inhibiting the differentiation of myoblasts. Although tetrandrine is a promising anticancer reagent, its inhibitory effects on skeletal muscle differentiation may limit its application in advanced cancer patients, who usually experience complications with sarcopenia or muscle injury. Therefore, great attention should be paid to the clinical use of tetrandrine.

## Data Availability

The raw data that support the findings of this study are available from the corresponding author, upon reasonable request.

## Supplementary Materials

The following supporting information can be downloaded at: https://www.mdpi.com/article/10.3390/ijms23158148/s1.
