# Supplementary material for: Tetrandrine Inhibits Skeletal Muscle Differentiation by Blocking Autophagic Flux
Source: Int J Mol Sci. 2022 Jul 24;23(15):8148. doi: 10.3390/ijms23158148 (PMC9331307; doi:10.3390/ijms23158148)
Supplement: Supplementary file 1 [file ijms-23-08148-s001.zip › ijms-1820189-supplementary.pdf]

Figure S1

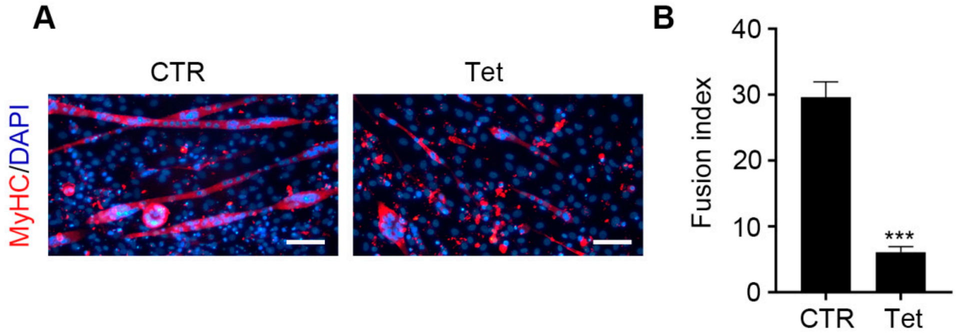

Figure S1 Tetrandrine inhibits myogenesis of primary mouse myoblasts. **A**: The myoblasts were differentiated for 5 d in DM with or without tetrandrine (2 μM) administration, immunostained with anti-MyHC (MF20) (red), the nuclei were counter stained with DAPI (blue). **B**: The fusion index in A were determined. n = 3, *** $p < 0.001$. Scal bars: 50 μm.
